# Altered Pre-mRNA Splicing Caused by a Novel Intronic Mutation c.1443+5G>A in the Dihydropyrimidinase (*DPYS*) Gene

**DOI:** 10.3390/ijms17010086

**Published:** 2016-01-12

**Authors:** Yoko Nakajima, Judith Meijer, Chunhua Zhang, Xu Wang, Tomomi Kondo, Tetsuya Ito, Doreen Dobritzsch, André B. P. Van Kuilenburg

**Affiliations:** 1 Department of Pediatrics, Fujita Health University School of Medicine, Toyoake 470-1192, Japan; tomomi-h@fujita-hu.ac.jp (T.K.); itotetsu@fujita-hu.ac.jp (T.I.); 2 Laboratory Genetic Metabolic Diseases, Academic Medical Center, Amsterdam 1105 AZ, The Netherlands; j.meijer@amc.uva.nl (J.M.); a.b.vankuilenburg@amc.uva.nl (A.B.P.V.K.); 3 Department of Research and Development, MILS International, Kanazawa 921-8105, Japan; mils@yacht.ocn.ne.jp; 4 Department of Neurology, Beijing Children’s Hospital Affiliated to Capital University of Medical Sciences, Beijing 100045, China; zfwx05@126.com; 5 Department of Chemistry-BMC, Uppsala University, Uppsala 75123, Sweden; doreen.dobritzsch@kemi.uu.se

**Keywords:** dihydropyrimidinase, *DPYS*, splicing, minigene

## Abstract

Dihydropyrimidinase (DHP) deficiency is an autosomal recessive disease caused by mutations in the *DPYS* gene. Patients present with highly elevated levels of dihydrouracil and dihydrothymine in their urine, blood and cerebrospinal fluid. The analysis of the effect of mutations in *DPYS* on pre-mRNA splicing is hampered by the fact that DHP is primarily expressed in liver and kidney cells. The minigene approach can detect mRNA splicing aberrations using cells that do not express the endogenous mRNA. We have used a minigene-based approach to analyze the effects of a presumptive pre-mRNA splicing mutation in two newly identified Chinese pediatric patients with DHP deficiency. Mutation analysis of *DPYS* showed that both patients were compound heterozygous for a novel intronic mutation c.1443+5G>A in intron 8 and a previously described missense mutation c.1001A>G (p.Q334R) in exon 6. Wild-type and the mutated minigene constructs, containing exons 7, 8 and 9 of *DPYS*, yielded different splicing products after expression in HEK293 cells. The c.1443+5G>A mutation resulted in altered pre-mRNA splicing of the *DPYS* minigene construct with full skipping of exon 8. Analysis of the DHP crystal structure showed that the deletion of exon 8 severely affects folding, stability and homooligomerization of the enzyme as well as disruption of the catalytic site. Thus, the analysis suggests that the c.1443+5G>A mutation results in aberrant splicing of the pre-mRNA encoding DHP, underlying the DHP deficiency in two unrelated Chinese patients.

## 1. Introduction

Dihydropyrimidinase (DHP, EC 3.5.2.2) is the second enzyme of the pyrimidine degradation pathway and catalyzes the ring opening of 5,6-dihydrouracil and 5,6-dihydrothymine. DHP deficiency (MIM 222748) is an autosomal recessive disease characterized by a large accumulation of dihydrouracil and dihydrothymine in urine, blood and cerebrospinal fluid [1,2]. The gene encoding DHP, *DPYS*, maps to chromosome 8q22 and comprises 10 exons spanning >80 kb of genomic DNA [3]. To date, 27 genetically confirmed complete DHP-deficient patients have been described, including six asymptomatic individuals who were identified by a screening program [1,3,4,5,6,7,8,9,10,11,12]. To date, at least 16 missense mutations, two nonsense mutations, two deletions, one insertion and one splice-site mutation have been reported [1,3,10]. Pre-mRNA splicing requires precise recognition of *cis*-acting sequences on the pre-mRNA by the spliceosome and additional RNA-binding factors and involves a vast network of RNA–RNA, RNA–protein and protein–protein interactions. Mis-regulation of splicing is associated with an increasing number of human pathologies, including neurodegenerative disorders, cancer and genetic diseases [13]. The minigene approach is able to detect mRNA splicing aberrations using cells that do not express the endogenous mRNA and is a powerful and simple molecular tool to analyze mutations that might affect splicing [14,15,16,17]. Here, we report two Chinese patients suffering from seizures who were found to have DHP deficiency caused by novel heterozygous *DPYS* mutations. The deleterious effect of an intronic mutation in *DPYS* that affected pre-mRNA splicing was demonstrated using the minigene approach.

## 2. Results

### 2.1. Urinary Concentration of Pyrimidine Metabolites Determined by HPLC-MS/MS

Urine screening for general inborn errors of metabolism by GCMS showed a gross elevation of dihydropyrimidines and, on this basis, DHP deficiency was suspected. Quantitation of relevant pyrimidines was performed by HPLC tandem-mass spectrometry. Urine samples of the two patients showed highly elevated levels of dihydrouracil and dihydrothymine and moderately elevated levels of uracil and thymine compared with controls (Table 1). The observed dihydropyrimidinuria in these patients strongly suggested DHP deficiency.

**Table 1:** Urinary concentrations of pyrimidine metabolites, as determined by HPLC-MS/MS.

Compound Patient (Age)	Uracil	Thymine	DHU ^a^	DHT ^b^	NCβ-Alanine ^c^	NCβ-AIB ^d^
1 (10 years)	42	26	237	138	<1	<0.5
2 (1.8 years)	115	85	539	309	<1	<1
Control (<3 years, *n* = 104)	11.8 ± 9.1	0.5 ± 0.6	6.3 ± 5.3	3.1 ± 2.1	11.0 ± 9.2	1.8 ± 2.3
Control (>3 years, *n* = 111)	7.0 ± 5.4	0.1 ± 0.3	2.1 ± 1.6	1.0 ± 0.7	2.8 ± 2.0	0.1 ± 0.4

Values are shown as µmol/mmol creatinine. Controls are indicated as mean ± SD. ^a^ Dihydrouracil; ^b^ Dihydrothymine; ^c^ *N*-carbamyl-β-alanine; ^d^ *N*-carbamyl-β-aminoisobutyric acid.

### 2.2. Genotype

Analysis of *DPYS* showed that both patients were compound heterozygous for the missense mutation c.1001A>G (p.Q334R) in exon 6 and a novel mutation c.1443+5G>A in intron 8. The mother of patient 1 and father of patient 2 were found to be heterozygous for c.1443+5G>A, while the father of patient 1 and mother of patient 2 were heterozygous for p.Q334R. To predict the effect of the c.1443+5G>A mutation on pre-mRNA splicing, *in silico* analysis was performed using three splice-site analysis programs. These analyses showed that the splice-donor scores for the sequence carrying the c.1443+5G>A mutation was significantly lower compared with those of the wild-type (WT) sequence: Splice Site Prediction by Neural Network; 0.28 *vs.* 0.99, MaxEntScan; 3.31 *vs.* 9.89, Human Splicing Finder; 81.82 *vs.* 93.98, respectively.

### 2.3. DPYS Minigene Expression

**Figure 1:**
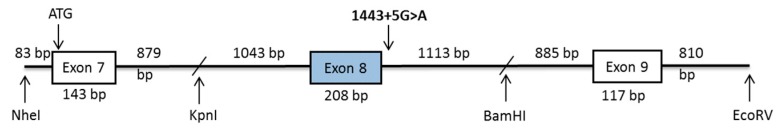
Schematic diagram of the DHP minigene construct containing exon 7, part of intron 7, exon 8, part of intron 8, exon 9 and part of intron 9.

**Figure 2:**
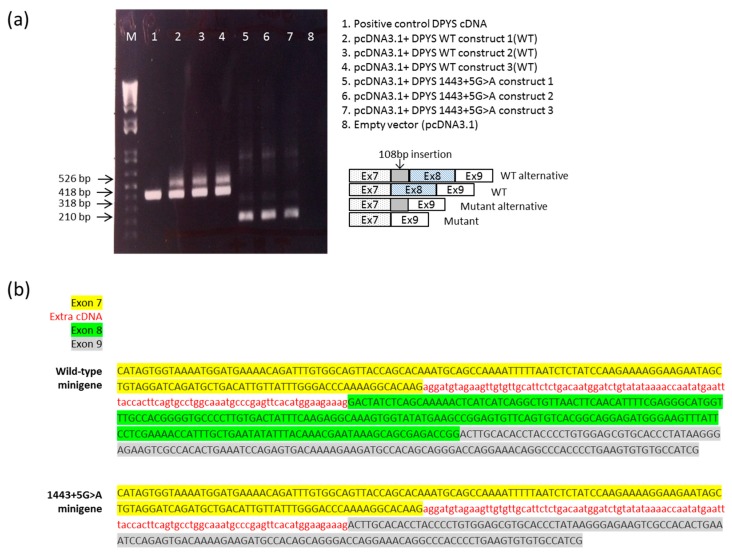
DHP minigene analysis. (**a**) Electrophoresis of RT-PCR amplification of the *DPYS* transcripts obtained from HEK293 cells transfected with the plasmid pcDNA3.1+ *DPYS* WT and pcDNA3.1+ *DPYS* 1443+5G>A. The experiment was performed at least three times independently; (**b**) Sequencing of the RT-PCR products from the wild-type and the mutant minigene.

A schematic representation of the *DPYS* minigene construct used to analyze the effect of the c.1443+5G>A mutation on pre-mRNA splicing is shown in Figure 1. The RT-PCR products of total RNA, which was extracted from HEK293 cells transfected with the minigene constructs, yielded different products for the WT and mutant *DPYS* minigene. RT-PCR products of the WT construct generated two splicing products of approximately 418 and 526 bp, with the 418-bp band being predominant (Figure 2a). Sequence analysis of the two PCR fragments of the WT construct showed that the predominant lower band corresponded to the expected normal splicing product of 418 bp containing exon 7, 8 and 9. The 526-bp product was generated by alternative splicing retaining a 108-bp sequence of intron 7 (Figure 2b). The c.1443+5G>A construct also produced two splicing products, with the 210-bp fragment being the most abundant product, whereas the 318-bp alternatively spliced product was barely visible (Figure 2a). The 210-bp band corresponded to a shorter RNA transcript, lacking the complete *DPYS* exon 8 (Figure 2b). The 318-bp transcript also lacked exon 8 but contained the additional sequence derived from intron 7.

### 2.4. Analysis of the Crystal Structure of Human DHP

**Figure 3:**
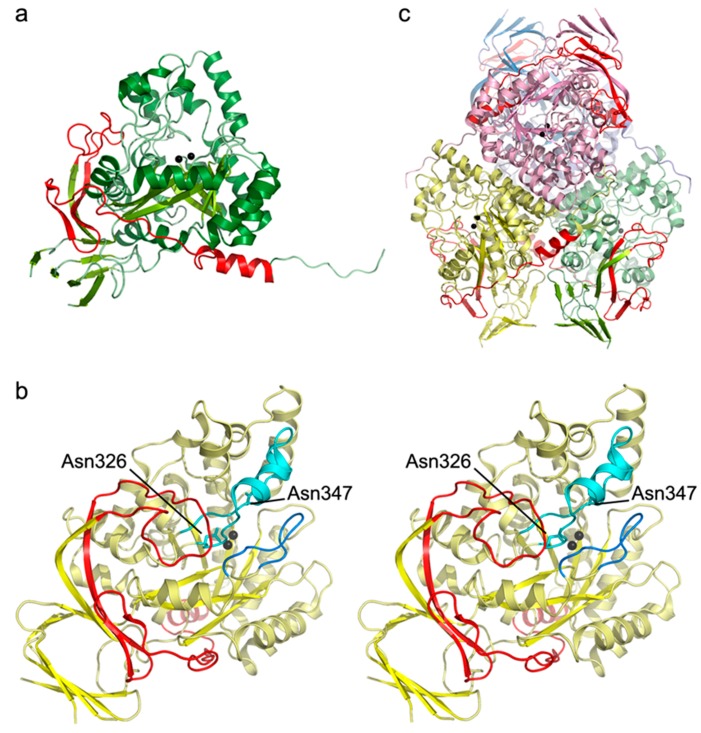
Location of the exon 8–encoded amino acids (in red) in the crystal structure of human dihydropyrimidinase (PDB-ID: 2VR2). (**a**) View of the dihydropyrimidinase subunit, with all but the exon 8–encoded amino acids shown in shades of green according to secondary structure. The zinc ions of the catalytic metal center are represented as black spheres; (**b**) Stereo view of the subunit from a different perspective compared to (**a**). The stereochemistry gate loop-1 (SGL-1) is depicted in blue, and the stretch of amino acids connecting the last barrel strand and barrel helix in cyan. Asp326 and Asn347 are labeled and shown as stick models in cyan; (**c**) The dihydropyrimidinase homotetramer, with the four subunits shown in green, yellow, pink and blue, respectively. The C-terminal helix (red) is wedged between the original and another subunit, while the following tail (not highlighted) is packed against the surface of a third subunit.

Exon 8 encodes amino acids 413–481 of the dihydropyrimidinase protein. Inspection of the deposited crystal structure of the human enzyme (unpublished, pdb accession code: 2VR2) revealed that these amino acids constitute three strands of one of the β-sheets in the β-sandwich domain, the C-terminal helix, as well as the loops connecting these elements (Figure 3a). Loss of the three strands would severely affect the folding and stability of the β-sandwich domain, and disrupt its interaction with the TIM barrel-like subunit core that harbors the active site. The long loop formed by residues 413–431, which connects the last strand encoded by exon 7 with the first missing strand, plays a particularly crucial role in these interactions (Figure 3b). It is packed against the barrel domain and contributes to correct and stable placement of the stretch of amino acids inserted between the last barrel strand and the helix that harbors the substrate-binding residue Asn347, and Asp326, serving both as a catalytic base and di-zinc center ligand. Furthermore, it also interacts with one of the stereochemistry gate loops (SGL-1, [18,19]), which are thought to determine the substrate specificity and enantioselectivity of dihydropyrimidinases. The splice-site mutation would also disturb homooligomerization because the deleted C-terminal helix and the following tail loop, which would be located elsewhere because of the exon skipping, significantly contribute to interface formation with two of the three other subunits in the homotetramer (Figure 3c).

## 3. Discussion

DHP deficiency is an inborn error of the pyrimidine degradation pathway and patients with complete DHP deficiency present with highly elevated levels of dihydropyrimidines in their urine. Clinical manifestations of this deficiency are variable, but neurological abnormalities, such as mental and motor developmental delay and seizures, are most frequently observed [10]. To date, the pathological mechanism underlying the neurological developmental abnormalities is still not known.

In this study, we showed that two DHP deficient patients were both compound heterozygous for the c.1001A>G (p.Q334R) and c.1443+5G>A mutations in *DPYS*. RNA of the patients was not available because DHP is primarily expressed in liver and kidney cells [20], which are inaccessible without invasive biopsy. The mutation p.Q334R has been reported in Japanese individuals previously, and functional analysis showed that the mutant enzyme possessed a significantly decreased activity compared to the WT enzyme [3]. The mutation c.1443+5G>A has not been reported before, and all three splice-site analysis programs used here predicted that the c.1443+5G>A mutation abolishes the normal splice-donor site of exon 8 and would result in aberrant splicing. The removal of introns is orchestrated by the spliceosome and during initial intron recognition, U1snRNP base-pairs with the 5′ splice-site consensus sequence (GURAGU) and U2snRNA base-pairs with the intron branch-point sequence [13]. The c.1443+5G>A in *DPYS* changes the 5ʹ splice-site consensus sequence from GUGAGU to GUGAAU and thus prevents the proper recognition of intron 8 by the spliceosome, resulting in the skipping of exon 8 immediately upstream of the mutated splice donor site during *DPYS* pre-mRNA splicing. Genetic variation within splice-sites and regulatory sequences frequently causes aberrant splicing in human hereditary diseases. Single nucleotide substitutions affecting the 5′ or the 3′ splice-site are the most common splicing mutations, which result in exon skipping, activation of a cryptic splice-site or, to a lesser extent, intron retention [13]. The minigene approach showed the correct splicing of exon 7, 8 and 9 of the mRNA transcribed from the wild-type construct, while splice products generated from cells transfected with the mutant construction revealed the skipping of exon 8. Interestingly, the three splice-site analysis programs also predicted the observed alternatively spliced transcript encompassing part of intron 7. The study of the crystal structure of human DHP provides a powerful tool to analyze the effect of amino acid substitutions and deletions on the function and stability of the enzyme. Analysis of the DHP crystal structure showed that the deletion of exon 8 severely affected folding, stability and homooligomerization of the enzyme as well as disruption of the catalytic site. Thus, the minigene analysis suggests that the c.1443+5G>A mutation results in aberrant splicing of the pre-mRNA encoding DHP and, as a consequence, a corrupted mutant DHP protein, underlying the DHP deficiency in two unrelated Chinese patients.

## 4. Methods

### 4.1. Patients

The first patient was a girl from Chinese non-consanguineous parents. She experienced an episode of loss of consciousness at the age of three months. After the incident, she showed normal development. When she was 10 years old, she presented with dizziness, unconsciousness and seizures. The second patient was a 22-month-old girl from Chinese non-consanguineous parents who presented with seizures and mental and motor developmental delay. 

### 4.2. Quantitative Analysis of Pyrimidines

The concentrations of uracil, thymine, dihydrouracil, dihydrothymine, *N*-carbamyl-β-alanine and *N*-carbamyl-β-aminoisobutyric acid in urine-soaked filter paper strips were determined using reversed-phase HPLC combined with electrospray tandem mass spectrometry [21,22].

### 4.3. PCR Amplification of Coding Exons of DPYS

DNA was isolated from blood spots using the QIAamp DNA Micro kit (QIAGEN, Hilden, Germany). Exons 2–9 of *DPYS* and flanking intronic regions were amplified using the primer sets as described previously [3]. For exon 1, two sets of primers, exon 1.1 (F-primer: 5′-ATATGCGGCCAGGTCATAAA-3′ and R-primer: 5′-ATGAAGGGGAACTGCATGTG-3′), and exon 1.2 (F-primer: 5′-ACGATGACTTCTCGGAGGTG-3′ and R-primer: 5′-CCCAGCGAAGAGAATCTGAG-3′) were used. All the forward primers had a -21M13 (5′-TGTAAAACGACGGCCAGT-3′) extension, whereas the reverse primers had an M13-Rev (5′-CAGGAAACAGCTATGACC-3′) extension at their 5′-ends. PCR products were separated on 1.5% agarose gels, visualized with ethidium bromide and used for direct sequencing. The *DPYS* sequence of the patient was compared to that observed in controls and the reference sequence of *DPYS* (Ref Seq NM_001385.2).

### 4.4. Construction of the Dihydropyrimidinaase Minigene

Three fragments of the human *DPYS* gene, containing exons 7, 8 and 9, respectively, were amplified by PCR using Phusion^®^ High-Fidelity DNA Polymerase (New England Biolabs, Ipswich, MA, USA) and primers engineered to contain restriction sites (Table 2). Amplification was carried out in 20-μL reaction mixtures containing 1× Phusion^®^ HF Buffer (New England Biolabs), 0.2 mM dNTPs, 0.5 μM of each primer and 0.4 U of Phusion^®^ DNA polymerase (New England Biolabs). After initial denaturation for 30 s at 98 °C, amplification was carried out for 30 cycles (10 s at 98 °C, 30 s at 67 °C, 1 min at 72 °C) with a final extension step of 10 min at 72 °C. Both the WT and mutant minigene construct were generated by amplification of fragment B of the patient’s genomic DNA. The resulting fragments were cloned into the pcDNA3.1Zeo vector using the restriction sites listed in Table 2, starting with fragment A, B and then C. Sequence analysis of the clones was carried out on a Applied Biosystems model 3730 automated DNA sequencer using the dye-terminator method (Applied Biosystems, Nieuwekerk a/d IJssel, The Netherlands) to distinguish the WT clone from the mutated clones.

**Table 2:** Minigene primer design.

PCR Product	Primer Sequence	Including Exon	Bp	Restriction Enzymes
Fragment A	F: 5′-tcatGCTAGCtgcaagtcttgtcattttatcg-3′	Exon 7	1268	*NheI Acc65I*
	R: 5′-gaaggctgcttgcttgctat–3′			
Fragment B	F: 5′-tcatGGTACCgtgggagcaaagctatgagg-3′	Exon 8	2382	*Acc65I BamHI*
	R: 5′-tcatGGATCCcatcaaaaggggaaagcaaa-3′			
Fragment C	F: 5′-tttcagatgtggtggtccaa-3′	Exon 9	2493	*BamHI EcoRV*
	R: 5′-gcattgaatcgcattccttt-3′			

### 4.5. Cell Culture and Transient Transfection

HEK293 cells were cultured in Dulbecco’s modified eagle’s medium with 4.5 g/L glucose, 25 mM Hepes, and 4 mM l-glutamine (Lonza, Basel, Switzerland), supplemented with 10% fetal bovine serum, 100 U/mL penicillin, 100 mg/mL streptomycin and 250 μg/mL fungizone at 37 °C in a humidified 5% CO_2_ incubator. For transient transfection, cell cultures were set up 24 hours before transfections in six-well plates. HEK293 cells were transfected with the pcDNA3.1Zeo-*DPYS* minigene (WT, mutant) using X-treme GENE HP DNA Transfection reagent (Roche, Basel, Switzerland). Two days after transfection, the cells were harvested and suspended in 1 mL TRIzol (Thermo Fisher, Waltham, MA, USA). The cell suspension was stored at −20 °C until use. The parental vector pcDNA3.1Zeo without insert was transfected as a negative control.

### 4.6. RNA Analysis of the Overexpressed pcDNA3.1Zeo-DPYS Minigene

Total RNA was extracted from the pellets of HEK293 cells containing the transiently expressed minigene with TRIzol reagent according to the manufacturer’s instructions. cDNA synthesis was performed using a Transcriptor First Strand cDNA Synthesis Kit (Roche). Briefly, the reaction was performed with 1 μg total RNA in a total volume of 20 μL, including the random primer. The reaction conditions were: 25 °C for 10 min, 55 °C for 30 min, and 80 °C for 5 min. cDNA was stored at −20 °C until use. *DPYS* cDNA was amplified using the primer set (F-primer: 5′-GATTTGTGGCAGTTACCAGC-3′, R-primer: 5′-CCTGCTGTGGCATCTTCTTT-3′). Amplification was carried out in a 25-μL reaction mixture containing 20 mM Tris–HCl (pH 8.4), 50 mM KCl, 1.5 mM MgCl_2_, 0.4 μM of each primer, 0.2 mM dNTPs, and 0.02 U of Platinum Taq polymerase (Thermo Fisher). After initial denaturation for 5 min at 95 °C, amplification was carried out for 30 cycles (30 s at 95 °C, 30 s at 55 °C, 60 s at 72 °C) with a final extension step of 10 min at 72 °C. PCR products were separated on 1.5% (*w*/*v*) agarose gels, visualized with ethidium bromide, treated with exoSAP-IT (USB, Cleveland, OH, USA) and used for direct sequencing.

## 5. Conclusions

DHP deficiency in our patients was caused by compound heterozygosity for *DPYS* missense mutation c.1001A>G (p.Q334R) and a novel exon-skipping splicing mutation c.1443+5G>A. Our findings in two unrelated Chinese patients suggested that DHP deficiency might be not as rare as generally considered. Although the minigene approach has been used successfully to investigate a synonymous mutation in the *UPB1* gene, the defect of the third enzyme of the pyrimidine degradation pathway [23], to the best of our knowledge, this is the first report showing the utility of this approach to study a mutation affecting pre-mRNA splicing in the *DPYS* gene. Our study demonstrated that the minigene strategy is an attractive approach to analyze the effects of potential splicing mutations in biochemically diagnosed DHP-deficient patients.

## References

[1] Van Kuilenburg A.B., Meijer J., Dobritzsch D., Meinsma R., Duran M., Lohkamp B., Zoetekouw L., Abeling N.G., van Tinteren H.L., Bosch A.M. (2007). Clinical, biochemical and genetic findings in two siblings with a dihydropyrimidinase deficiency. Mol. Genet. Metab..

[2] Jurecka A. (2009). Inborn errors of purine and pyrimidine metabolism. J. Inherit. Metab Dis..

[3] Hamajima N., Kouwaki M., Vreken P., Matsuda K., Sumi S., Imaeda M., Ohba S., Kidouchi K., Nonaka M., Sasaki M. (1998). Dihydropyrimidinase deficiency: Structural organization, chromosomal localization, and mutation analysis of the human dihydropyrimidinase gene. Am. J. Hum. Genet..

[4] Duran M., Rovers P., de Bree P.K., Schreuder C.H., Beukenhorst H., Dorland L., Berger R. (1991). Dihydropyrimidinuria: A new inborn error of pyrimidine metabolism. J. Inherit. Metab. Dis..

[5] Assmann B., Hoffmann G.F., Wagner L., Brautigam C., Seyberth H.W., Duran M., van Kuilenburg A.B., Wevers R., van Gennip A.H. (1997). Dihydropyrimidinase deficiency and congenital microvillous atrophy: Coincidence or genetic relation?. J. Inherit. Metab. Dis..

[6] Duran M., Rovers P., de Bree P.K., Schreuder C.H., Beukenhorst H., Dorland L., Berger R. (1990). Dihydropyrimidinuria. Lancet.

[7] Henderson M.J., Ward K., Simmonds H.A., Duley J.A., Davies P.M. (1993). Dihydropyrimidinase deficiency presenting in infancy with severe developmental delay. J. Inherit. Metab. Dis..

[8] Putman C.W., Rotteveel J.J., Wevers R.A., van Gennip A.H., Bakkeren J.A., de Abreu R.A. (1997). Dihydropyrimidinase deficiency, a progressive neurological disorder?. Neuropediatrics.

[9] Sumi S., Imaeda M., Kidouchi K., Ohba S., Hamajima N., Kodama K., Togari H., Wada Y. (1998). Population and family studies of dihydropyrimidinuria: Prevalence, inheritance mode, and risk of fluorouracil toxicity. Am. J. Med. Genet..

[10] Van Kuilenburg A.B., Dobritzsch D., Meijer J., Meinsma R., Benoist J.F., Assmann B., Schubert S., Hoffmann G.F., Duran M., de Vries M.C. (2010). Dihydropyrimidinase deficiency: Phenotype, genotype and structural consequences in 17 patients. Biochim. Biophys. Acta.

[11] Yeung C.W., Yau M.M., Ma C.K., Siu T.S., Tam S., Lam C.W. (2013). Diagnosis of dihydropyrimidinase deficiency in a Chinese boy with dihydropyrimidinuria. Hong Kong Med. J..

[12] Hiratsuka M., Yamashita H., Akai F., Hosono H., Hishinuma E., Hirasawa N., Mori T. (2015). Genetic polymorphisms of dihydropyrimidinase in a Japanese patient with capecitabine-induced toxicity. PLoS ONE.

[13] Daguenet E., Dujardin G., Valcarcel J. (2015). The pathogenicity of splicing defects: Mechanistic insights into pre-mRNA processing inform novel therapeutic approaches. EMBO Rep..

[14] Gaildrat P., Killian A., Martins A., Tournier I., Frebourg T., Tosi M. (2010). Use of splicing reporter minigene assay to evaluate the effect on splicing of unclassified genetic variants. Methods Mol. Biol..

[15] Gamez-Pozo A., Palacios I., Kontic M., Menendez I., Camino I., Garcia-Miguel P., Abelairas J., Pestana A., Alonso J. (2007). Pathogenic validation of unique germline intronic variants of RB1 in retinoblastoma patients using minigenes. Hum. Mutat..

[16] Giorgi G., Casarin A., Trevisson E., Dona M., Cassina M., Graziano C., Picci L., Clementi M., Salviati L. (2015). Validation of CFTR intronic variants identified during cystic fibrosis population screening by a minigene splicing assay. Clin. Chem. Lab. Med..

[17] Nielsen K.B., Sorensen S., Cartegni L., Corydon T.J., Doktor T.K., Schroeder L.D., Reinert L.S., Elpeleg O., Krainer A.R., Gregersen N. (2007). Seemingly neutral polymorphic variants may confer immunity to splicing-inactivating mutations: a synonymous SNP in exon 5 of MCAD protects from deleterious mutations in a flanking exonic splicing enhancer. Am. J. Hum. Genet..

[18] Lohkamp B., Andersen B., Piskur J., Dobritzsch D. (2006). The crystal structures of dihydropyrimidinases reaffirm the close relationship between cyclic amidohydrolases and explain their substrate specificity. J. Biol. Chem..

[19] Cheon Y.H., Kim H.S., Han K.H., Abendroth J., Niefind K., Schomburg D., Wang J., Kim Y. (2002). Crystal structure of d-hydantoinase from Bacillus stearothermophilus: insight into the stereochemistry of enantioselectivity. Biochemistry.

[20] Van Kuilenburg A.B., van Lenthe H., van Gennip A.H. (2006). Activity of pyrimidine degradation enzymes in normal tissues. Nucleosides Nucleotides Nucleic Acids.

[21] Van Kuilenburg A.B., van Lenthe H., van Cruchten A., Kulik W. (2004). Quantification of 5,6-dihydrouracil by HPLC-electrospray tandem mass spectrometry. Clin Chem..

[22] Van Lenthe H., van Kuilenburg A.B., Ito T., Bootsma A.H., van Cruchten A., Wada Y., van Gennip A.H. (2000). Defects in pyrimidine degradation identified by HPLC-electrospray tandem mass spectrometry of urine specimens or urine-soaked filter paper strips. Clin. Chem..

[23] Meijer J., Nakajima Y., Zhang C., Meinsma R., Ito T., van Kuilenburg A.B. (2013). Identification of a novel synonymous mutation in the human beta-Ureidopropionase Gene UPB1 affecting pre-mRNA splicing. Nucleosides Nucleotides Nucleic Acids.

